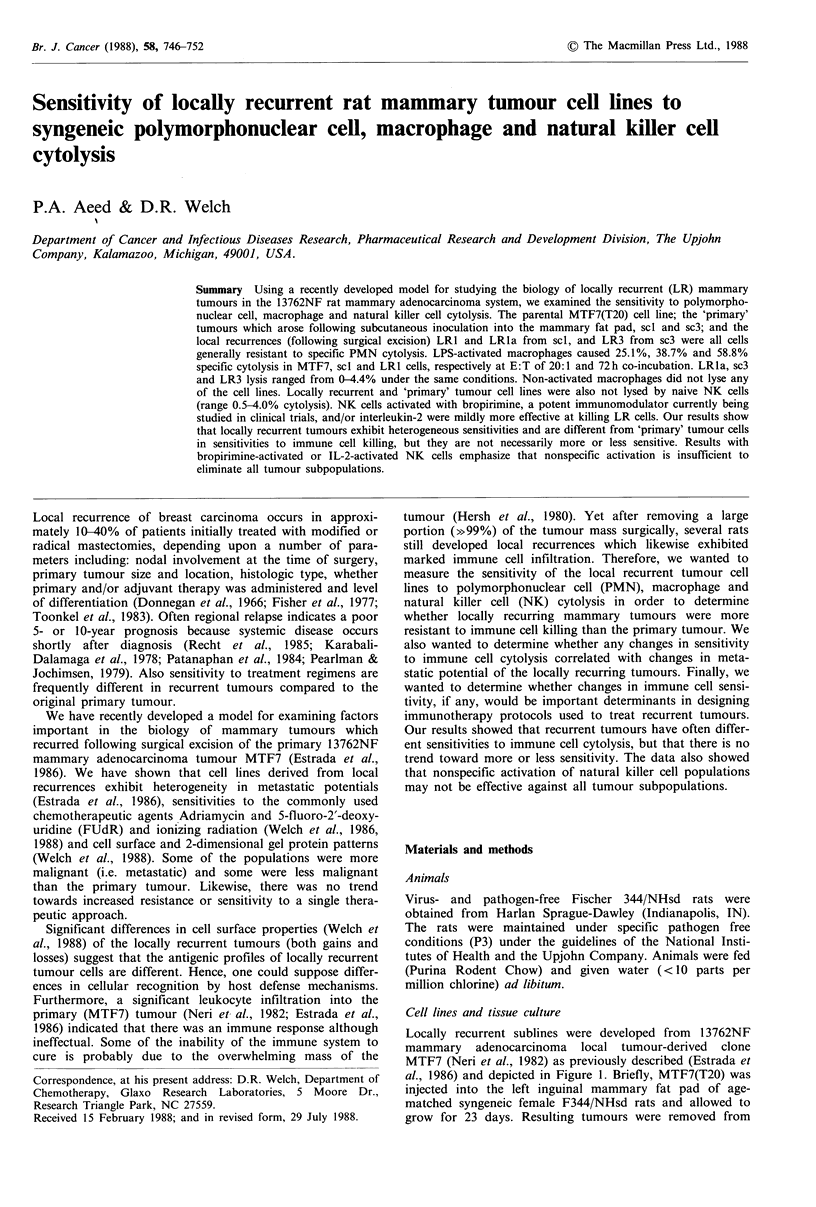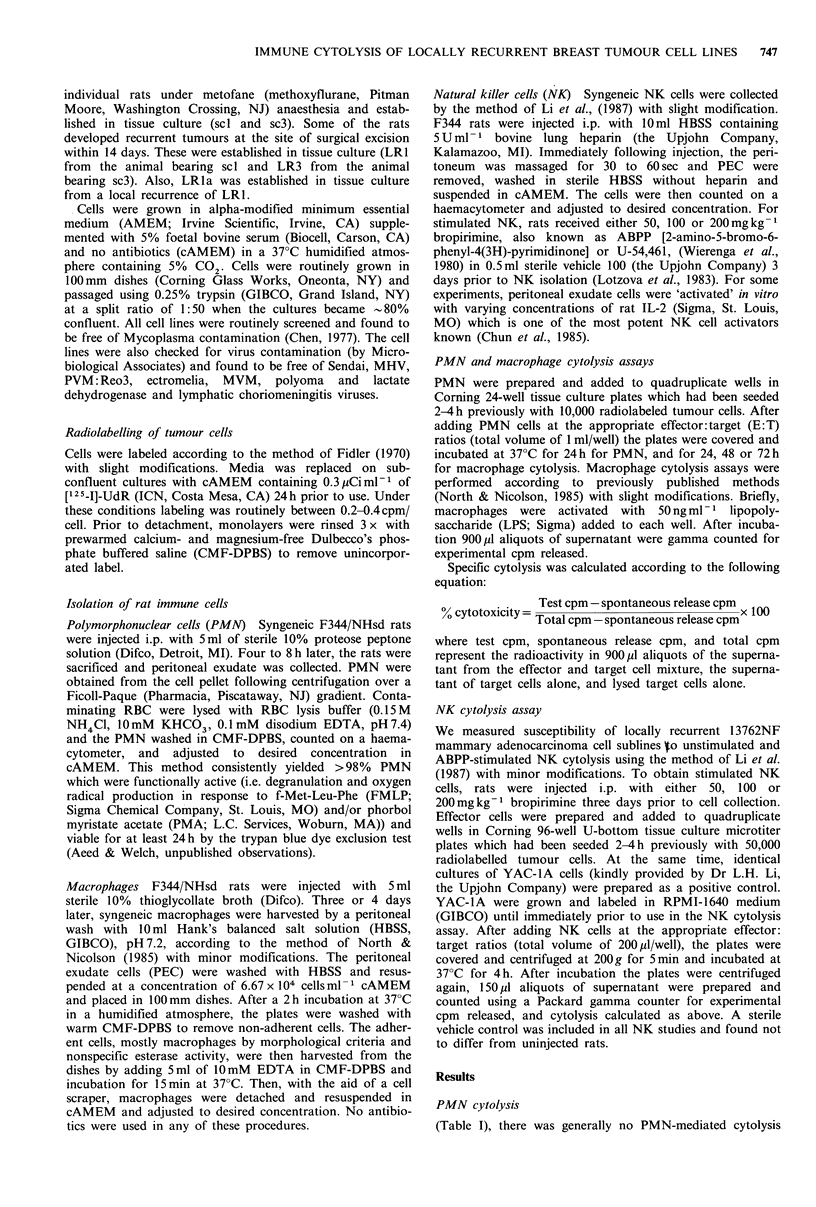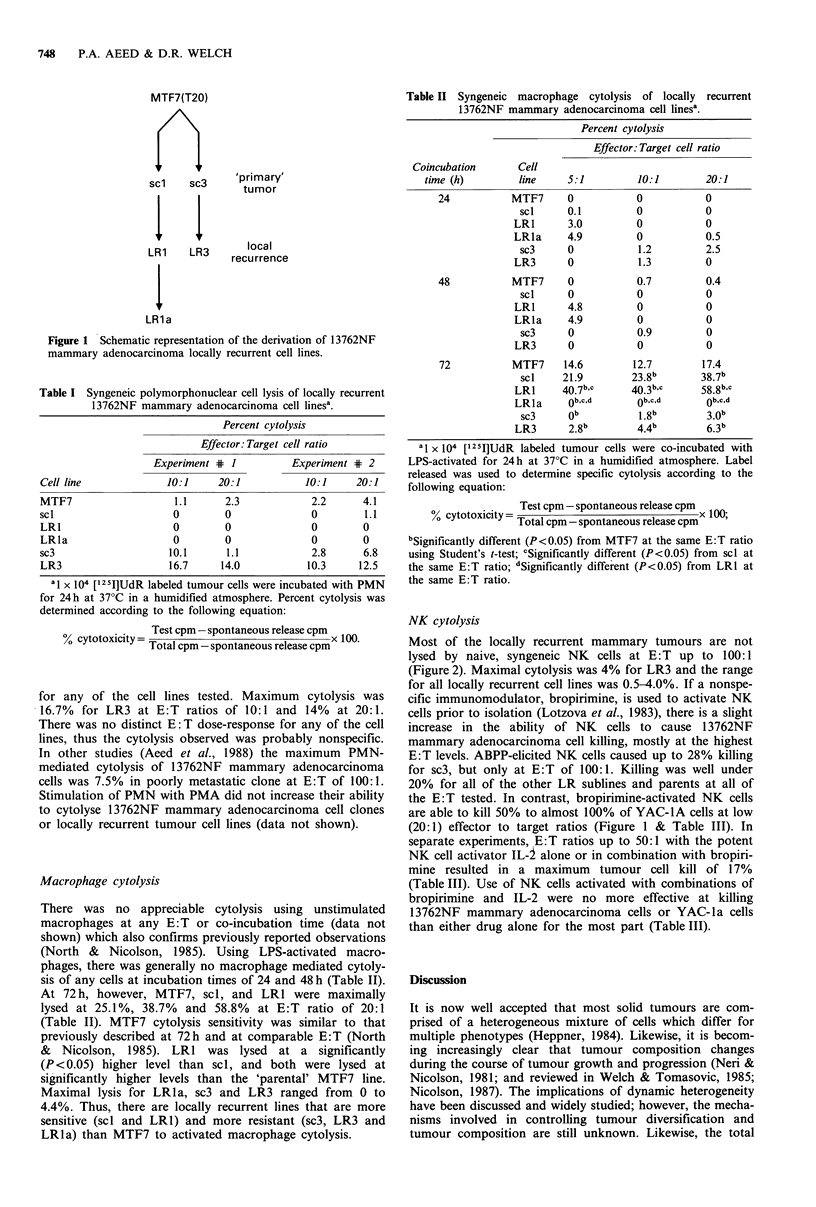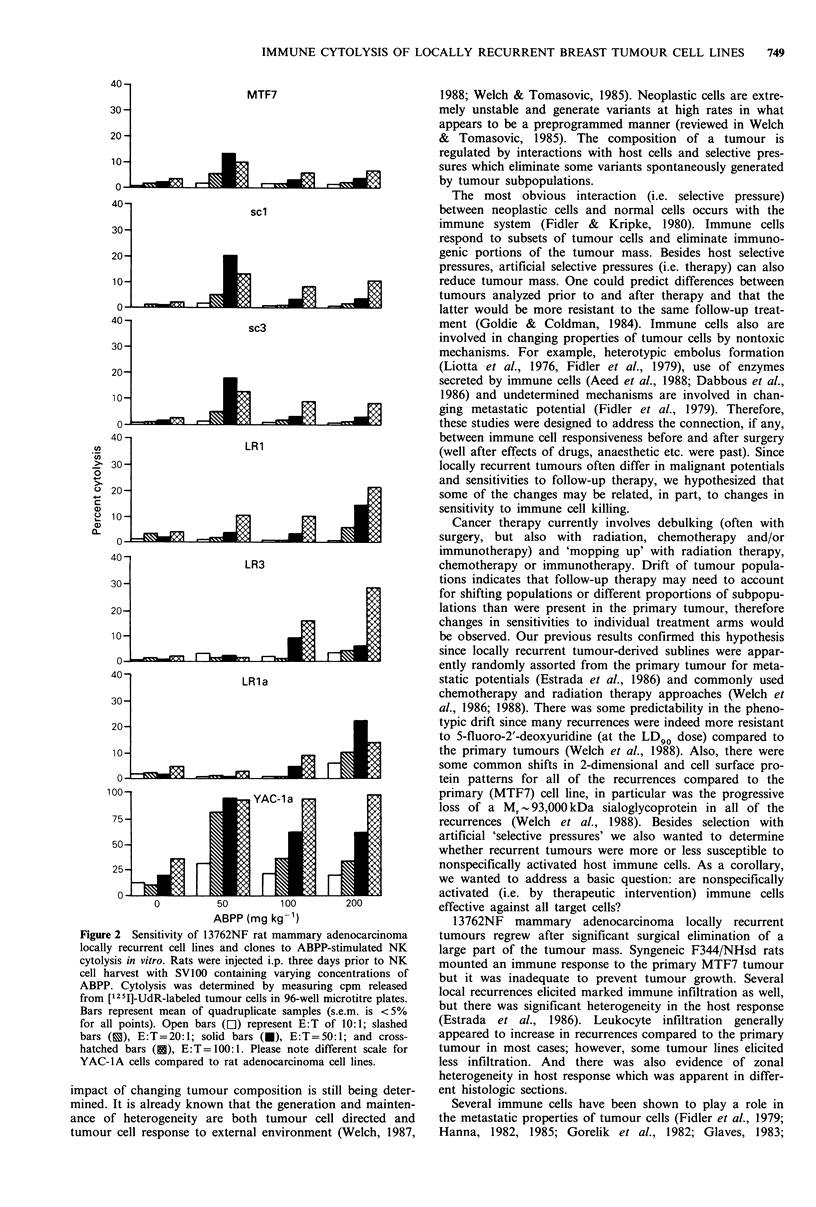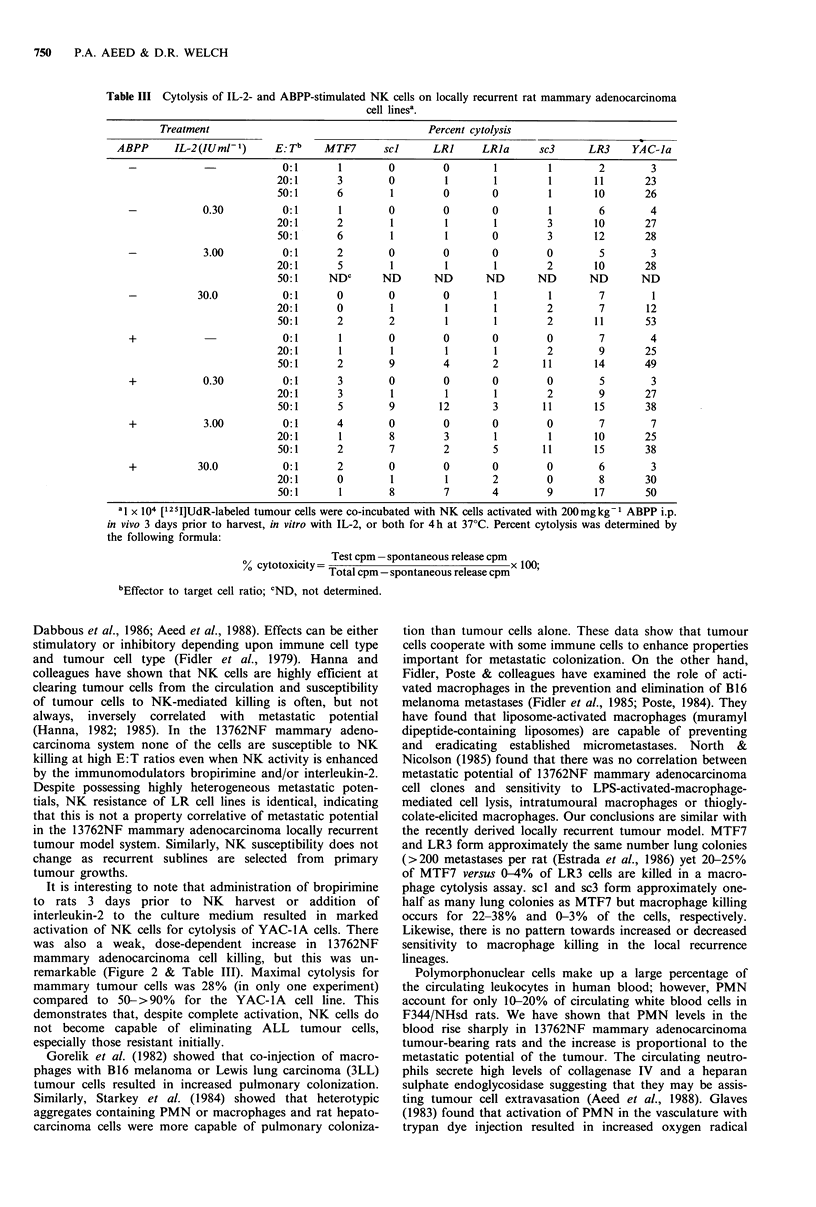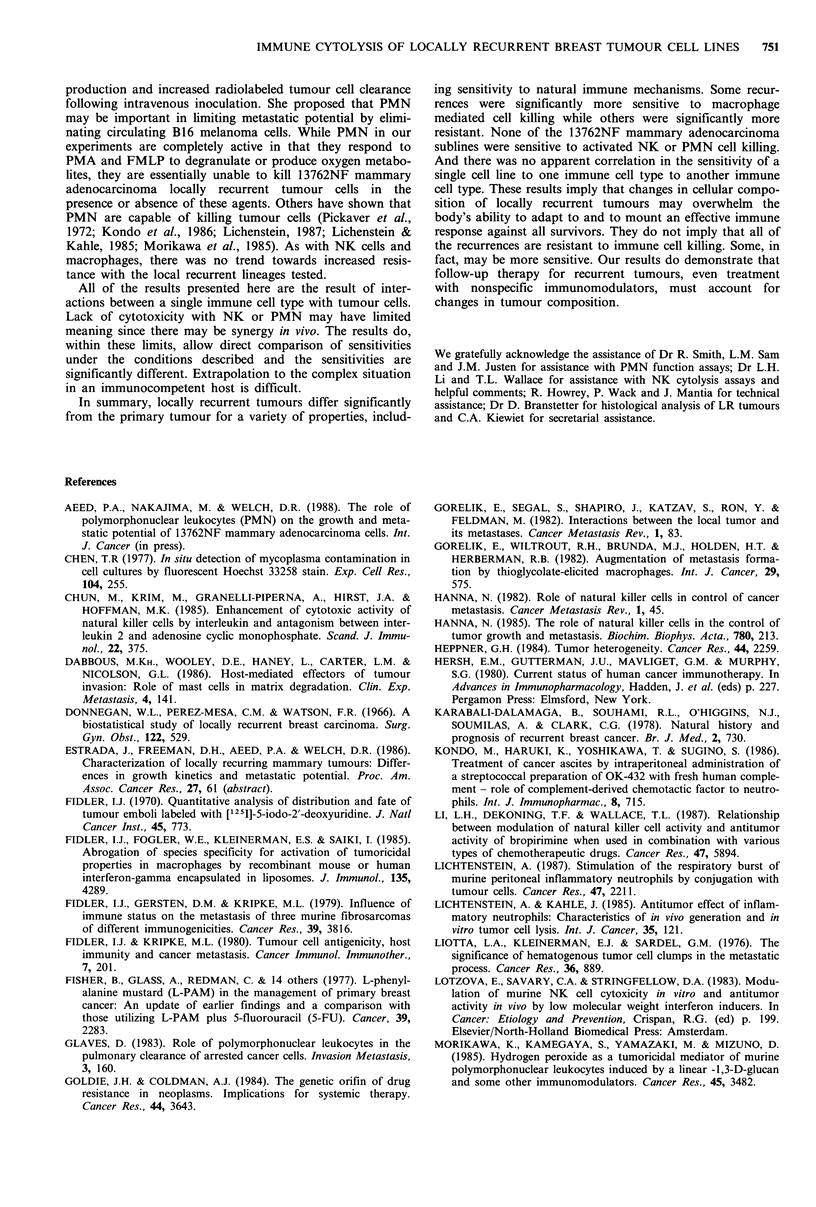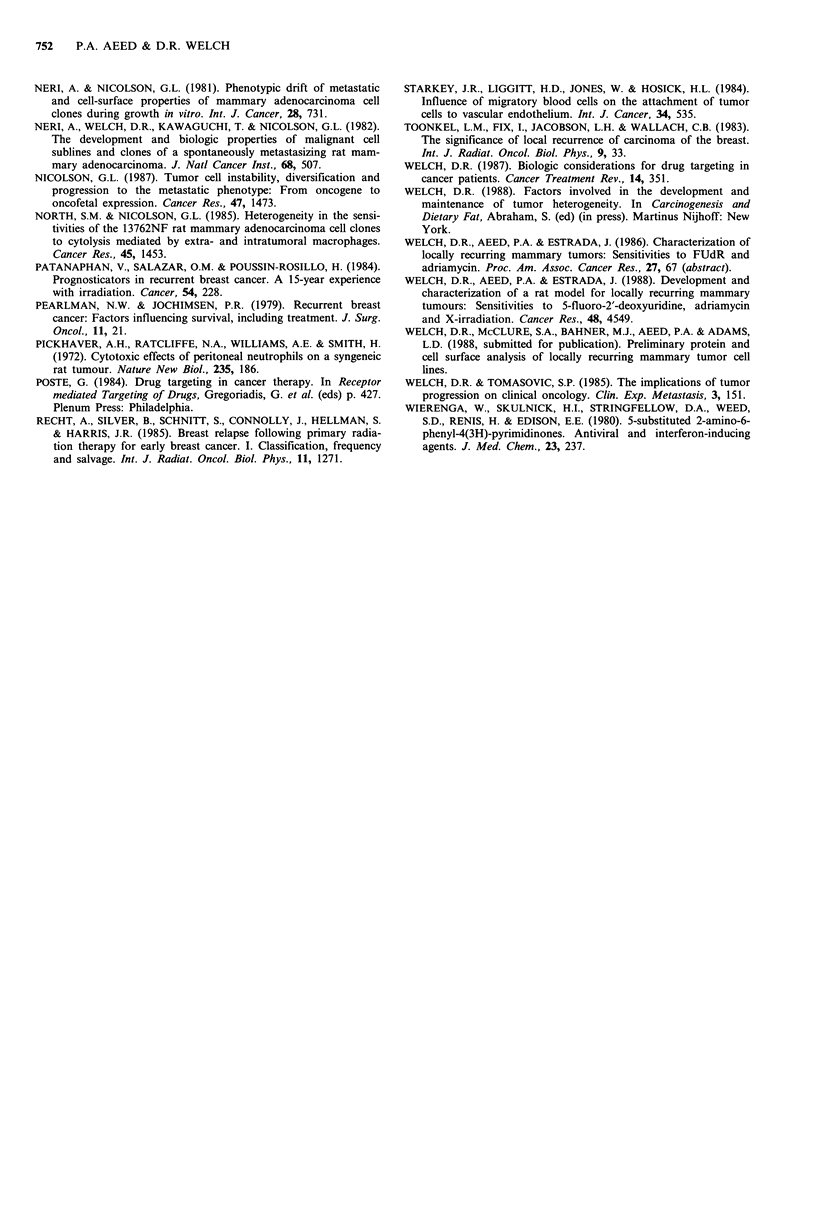# Sensitivity of locally recurrent rat mammary tumour cell lines to syngeneic polymorphonuclear cell, macrophage and natural killer cell cytolysis.

**DOI:** 10.1038/bjc.1988.302

**Published:** 1988-12

**Authors:** P. A. Aeed, D. R. Welch

**Affiliations:** Department of Cancer and Infectious Diseases Research, Upjohn Company, Kalamazoo, Michigan 49001.

## Abstract

Using a recently developed model for studying the biology of locally recurrent (LR) mammary tumours in the 13762NF rat mammary adenocarcinoma system, we examined the sensitivity to polymorphonuclear cell, macrophage and natural killer cell cytolysis. The parental MTF7(T20) cell line; the 'primary' tumours which arose following subcutaneous inoculation into the mammary fat pad, sc1 and sc3; and the local recurrences (following surgical excision) LR1 and LR1a from sc1, and LR3 from sc3 were all cells generally resistant to specific PMN cytolysis. LPS-activated macrophages caused 25.1%, 38.7% and 58.8% specific cytolysis in MTF7, sc1 and LR1 cells, respectively at E:T of 20:1 and 72 h co-incubation. LR1a, sc3 and LR3 lysis ranged from 0-4.4% under the same conditions. Non-activated macrophages did not lyse any of the cell lines. Locally recurrent and 'primary' tumour cell lines were also not lysed by naive NK cells (range 0.5-4.0% cytolysis). NK cells activated with bropirimine, a potent immunomodulator currently being studied in clinical trials, and/or interleukin-2 were mildly more effective at killing LR cells. Our results show that locally recurrent tumours exhibit heterogeneous sensitivities and are different from 'primary' tumour cells in sensitivities to immune cell killing, but they are not necessarily more or less sensitive. Results with bropirimine-activated or IL-2-activated NK cells emphasize that nonspecific activation is insufficient to eliminate all tumour subpopulations.


*Br. J. Cancer* (1988), **58**, 746–752 © The Macmillan Press Ltd., 1988

### Sensitivity of locally recurrent rat mammary tumour cell lines to syngeneic polymorphonuclear cell, macrophage and natural killer cell cytolysis

P.A. Aeed & D.R. Welch

*Department of Cancer and Infectious Diseases Research, Pharmaceutical Research and Development Division, The Upjohn Company, Kalamazoo, Michigan, 49001, USA.*

**Summary** Using a recently developed model for studying the biology of locally recurrent (LR) mammary tumours in the 13762NF rat mammary adenocarcinoma system, we examined the sensitivity to polymorphonuclear cell, macrophage and natural killer cell cytolysis. The parental MTF7(T20) cell line; the 'primary' tumours which arose following subcutaneous inoculation into the mammary fat pad, scl and sc3; and the local recurrences (following surgical excision) LR1 and LR1a from scl, and LR3 from sc3 were all cells generally resistant to specific PMN cytolysis. LPS-activated macrophages caused 25.1%, 38.7% and 58.8% specific cytolysis in MTF7, scl and LR1 cells, respectively at E:T of 20:1 and 72h co-incubation. LR1a, sc3 and LR3 lysis ranged from 0–4.4% under the same conditions. Non-activated macrophages did not lyse any of the cell lines. Locally recurrent and 'primary' tumour cell lines were also not lysed by naive NK cells (range 0.5–4.0% cytolysis). NK cells activated with bropirimine, a potent immunomodulator currently being studied in clinical trials, and/or interleukin-2 were mildly more effective at killing LR cells. Our results show that locally recurrent tumours exhibit heterogeneous sensitivities and are different from 'primary' tumour cells in sensitivities to immune cell killing, but they are not necessarily more or less sensitive. Results with bropirimine-activated or IL-2-activated NK cells emphasize that nonspecific activation is insufficient to eliminate all tumour subpopulations.

Local recurrence of breast carcinoma occurs in approximately 10–40% of patients initially treated with modified or radical mastectomies, depending upon a number of parameters including: nodal involvement at the time of surgery, primary tumour size and location, histologic type, whether primary and/or adjuvant therapy was administered and level of differentiation (Donnegan *et al.*, 1966; Fisher *et al.*, 1977; Toonkel *et al.*, 1983). Often regional relapse indicates a poor 5- or 10-year prognosis because systemic disease occurs shortly after diagnosis (Recht *et al.*, 1985; Karabali-Dalamaga *et al.*, 1978; Patanaphan *et al.*, 1984; Pearlman & Jochimsen, 1979). Also sensitivity to treatment regimens are frequently different in recurrent tumours compared to the original primary tumour.

We have recently developed a model for examining factors important in the biology of mammary tumours which recurred following surgical excision of the primary 13762NF mammary adenocarcinoma tumour MTF7 (Estrada *et al.*, 1986). We have shown that cell lines derived from local recurrences exhibit heterogeneity in metastatic potentials (Estrada *et al.*, 1986), sensitivities to the commonly used chemotherapeutic agents Adriamycin and 5-fluoro-2′-deoxyuridine (FUdR) and ionizing radiation (Welch *et al.*, 1986, 1988) and cell surface and 2-dimensional gel protein patterns (Welch *et al.*, 1988). Some of the populations were more malignant (i.e. metastatic) and some were less malignant than the primary tumour. Likewise, there was no trend towards increased resistance or sensitivity to a single therapeutic approach.

Significant differences in cell surface properties (Welch *et al.*, 1988) of the locally recurrent tumours (both gains and losses) suggest that the antigenic profiles of locally recurrent tumour cells are different. Hence, one could suppose differences in cellular recognition by host defense mechanisms. Furthermore, a significant leukocyte infiltration into the primary (MTF7) tumour (Neri *et al.*, 1982; Estrada *et al.*, 1986) indicated that there was an immune response although ineffectual. Some of the inability of the immune system to cure is probably due to the overwhelming mass of the tumour (Hersh *et al.*, 1980). Yet after removing a large portion (≫99%) of the tumour mass surgically, several rats still developed local recurrences which likewise exhibited marked immune cell infiltration. Therefore, we wanted to measure the sensitivity of the local recurrent tumour cell lines to polymorphonuclear cell (PMN), macrophage and natural killer cell (NK) cytolysis in order to determine whether locally recurring mammary tumours were more resistant to immune cell killing than the primary tumour. We also wanted to determine whether any changes in sensitivity to immune cell cytolysis correlated with changes in metastatic potential of the locally recurring tumours. Finally, we wanted to determine whether changes in immune cell sensitivity, if any, would be important determinants in designing immunotherapy protocols used to treat recurrent tumours. Our results showed that recurrent tumours have often different sensitivities to immune cell cytolysis, but that there is no trend toward more or less sensitivity. The data also showed that nonspecific activation of natural killer cell populations may not be effective against all tumour subpopulations.

### Materials and methods

### *Animals*

Virus- and pathogen-free Fischer 344/NHsd rats were obtained from Harlan Sprague-Dawley (Indianapolis, IN). The rats were maintained under specific pathogen free conditions (P3) under the guidelines of the National Institutes of Health and the Upjohn Company. Animals were fed (Purina Rodent Chow) and given water (<10 parts per million chlorine) *ad libitum.*

### *Cell lines and tissue culture*

Locally recurrent sublines were developed from 13762NF mammary adenocarcinoma local tumour-derived clone MTF7 (Neri *et al.*, 1982) as previously described (Estrada *et al.*, 1986) and depicted in Figure 1. Briefly, MTF7(T20) was injected into the left inguinal mammary fat pad of age-matched syngeneic female F344/NHsd rats and allowed to grow for 23 days. Resulting tumours were removed from

Correspondence, at his present address: D.R. Welch, Department of Chemotherapy, Glaxo Research Laboratories, 5 Moore Dr., Research Triangle Park, NC 27559.
Received 15 February 1988; and in revised form, 29 July 1988.

individual rats under metofane (methoxyflurane, Pitman Moore, Washington Crossing, NJ) anaesthesia and established in tissue culture (sc1 and sc3). Some of the rats developed recurrent tumours at the site of surgical excision within 14 days. These were established in tissue culture (LR1 from the animal bearing sc1 and LR3 from the animal bearing sc3). Also, LR1a was established in tissue culture from a local recurrence of LR1.

Cells were grown in alpha-modified minimum essential medium (AMEM; Irvine Scientific, Irvine, CA) supplemented with 5% foetal bovine serum (Biocell, Carson, CA) and no antibiotics (cAMEM) in a 37°C humidified atmosphere containing 5% $CO_2$. Cells were routinely grown in 100 mm dishes (Corning Glass Works, Oneonta, NY) and passaged using 0.25% trypsin (GIBCO, Grand Island, NY) at a split ratio of 1:50 when the cultures became ~80% confluent. All cell lines were routinely screened and found to be free of Mycoplasma contamination (Chen, 1977). The cell lines were also checked for virus contamination (by Microbiological Associates) and found to be free of Sendai, MHV, PVM:Reo3, ectromelia, MVM, polyoma and lactate dehydrogenase and lymphatic choriomeningitis viruses.

*Radiolabelling of tumour cells*

Cells were labeled according to the method of Fidler (1970) with slight modifications. Media was replaced on subconfluent cultures with cAMEM containing 0.3 $\mu$Ci ml$^{-1}$ of [$^{125}$-I]-UdR (ICN, Costa Mesa, CA) 24 h prior to use. Under these conditions labeling was routinely between 0.2–0.4 cpm/cell. Prior to detachment, monolayers were rinsed 3 × with prewarmed calcium- and magnesium-free Dulbecco's phosphate buffered saline (CMF-DPBS) to remove unincorporated label.

*Isolation of rat immune cells*

*Polymorphonuclear cells (PMN)* Syngeneic F344/NHsd rats were injected i.p. with 5 ml of sterile 10% proteose peptone solution (Difco, Detroit, MI). Four to 8 h later, the rats were sacrificed and peritoneal exudate was collected. PMN were obtained from the cell pellet following centrifugation over a Ficoll-Paque (Pharmacia, Piscataway, NJ) gradient. Contaminating RBC were lysed with RBC lysis buffer (0.15 M $NH_4Cl$, 10 mM $KHCO_3$, 0.1 mM disodium EDTA, pH 7.4) and the PMN washed in CMF-DPBS, counted on a haemacytometer, and adjusted to desired concentration in cAMEM. This method consistently yielded >98% PMN which were functionally active (i.e. degranulation and oxygen radical production in response to f-Met-Leu-Phe (FMLP; Sigma Chemical Company, St. Louis, MO) and/or phorbol myristate acetate (PMA; L.C. Services, Woburn, MA)) and viable for at least 24 h by the trypan blue dye exclusion test (Aeed & Welch, unpublished observations).

*Macrophages* F344/NHsd rats were injected with 5 ml sterile 10% thioglycollate broth (Difco). Three or 4 days later, syngeneic macrophages were harvested by a peritoneal wash with 10 ml Hank's balanced salt solution (HBSS, GIBCO), pH 7.2, according to the method of North & Nicolson (1985) with minor modifications. The peritoneal exudate cells (PEC) were washed with HBSS and resuspended at a concentration of $6.67 \times 10^4$ cells ml$^{-1}$ cAMEM and placed in 100 mm dishes. After a 2 h incubation at 37°C in a humidified atmosphere, the plates were washed with warm CMF-DPBS to remove non-adherent cells. The adherent cells, mostly macrophages by morphological criteria and nonspecific esterase activity, were then harvested from the dishes by adding 5 ml of 10 mM EDTA in CMF-DPBS and incubation for 15 min at 37°C. Then, with the aid of a cell scraper, macrophages were detached and resuspended in cAMEM and adjusted to desired concentration. No antibiotics were used in any of these procedures.

*Natural killer cells (NK)* Syngeneic NK cells were collected by the method of Li *et al.*, (1987) with slight modification. F344 rats were injected i.p. with 10 ml HBSS containing 5 U ml$^{-1}$ bovine lung heparin (the Upjohn Company, Kalamazoo, MI). Immediately following injection, the peritoneum was massaged for 30 to 60 sec and PEC were removed, washed in sterile HBSS without heparin and suspended in cAMEM. The cells were then counted on a haemacytometer and adjusted to desired concentration. For stimulated NK, rats received either 50, 100 or 200 mg kg$^{-1}$ bropirimine, also known as ABPP [2-amino-5-bromo-6-phenyl-4(3H)-pyrimidinone] or U-54,461, (Wierenga *et al.*, 1980) in 0.5 ml sterile vehicle 100 (the Upjohn Company) 3 days prior to NK isolation (Lotzova *et al.*, 1983). For some experiments, peritoneal exudate cells were 'activated' *in vitro* with varying concentrations of rat IL-2 (Sigma, St. Louis, MO) which is one of the most potent NK cell activators known (Chun *et al.*, 1985).

*PMN and macrophage cytolysis assays*

PMN were prepared and added to quadruplicate wells in Corning 24-well tissue culture plates which had been seeded 2–4 h previously with 10,000 radiolabeled tumour cells. After adding PMN cells at the appropriate effector:target (E:T) ratios (total volume of 1 ml/well) the plates were covered and incubated at 37°C for 24 h for PMN, and for 24, 48 or 72 h for macrophage cytolysis. Macrophage cytolysis assays were performed according to previously published methods (North & Nicolson, 1985) with slight modifications. Briefly, macrophages were activated with 50 ng ml$^{-1}$ lipopolysaccharide (LPS; Sigma) added to each well. After incubation 900 $\mu$l aliquots of supernatant were gamma counted for experimental cpm released.

Specific cytolysis was calculated according to the following equation:

$$\%\ \text{cytotoxicity} = \frac{\text{Test cpm} - \text{spontaneous release cpm}}{\text{Total cpm} - \text{spontaneous release cpm}} \times 100$$

where test cpm, spontaneous release cpm, and total cpm represent the radioactivity in 900 $\mu$l aliquots of the supernatant from the effector and target cell mixture, the supernatant of target cells alone, and lysed target cells alone.

*NK cytolysis assay*

We measured susceptibility of locally recurrent 13762NF mammary adenocarcinoma cell sublines to unstimulated and ABPP-stimulated NK cytolysis using the method of Li *et al.* (1987) with minor modifications. To obtain stimulated NK cells, rats were injected i.p. with either 50, 100 or 200 mg kg$^{-1}$ bropirimine three days prior to cell collection. Effector cells were prepared and added to quadruplicate wells in Corning 96-well U-bottom tissue culture microtiter plates which had been seeded 2–4 h previously with 50,000 radiolabelled tumour cells. At the same time, identical cultures of YAC-1A cells (kindly provided by Dr L.H. Li, the Upjohn Company) were prepared as a positive control. YAC-1A were grown and labeled in RPMI-1640 medium (GIBCO) until immediately prior to use in the NK cytolysis assay. After adding NK cells at the appropriate effector: target ratios (total volume of 200 $\mu$l/well), the plates were covered and centrifuged at 200 *g* for 5 min and incubated at 37°C for 4 h. After incubation the plates were centrifuged again, 150 $\mu$l aliquots of supernatant were prepared and counted using a Packard gamma counter for experimental cpm released, and cytolysis calculated as above. A sterile vehicle control was included in all NK studies and found not to differ from uninjected rats.

### Results

*PMN cytolysis*

(Table I), there was generally no PMN-mediated cytolysis

**Figure 1** Schematic representation of the derivation of 13762NF mammary adenocarcinoma locally recurrent cell lines.

**Table I** Syngeneic polymorphonuclear cell lysis of locally recurrent 13762NF mammary adenocarcinoma cell lines[a].

| | *Percent cytolysis* | | | |
|---|---|---|---|---|
| | *Effector: Target cell ratio* | | | |
| | *Experiment # 1* | | *Experiment # 2* | |
| *Cell line* | *10:1* | *20:1* | *10:1* | *20:1* |
| MTF7 | 1.1 | 2.3 | 2.2 | 4.1 |
| scl | 0 | 0 | 0 | 1.1 |
| LR1 | 0 | 0 | 0 | 0 |
| LR1a | 0 | 0 | 0 | 0 |
| sc3 | 10.1 | 1.1 | 2.8 | 6.8 |
| LR3 | 16.7 | 14.0 | 10.3 | 12.5 |

[a]$1 \times 10^4$ [$^{125}$I]UdR labeled tumour cells were incubated with PMN for 24h at 37°C in a humidified atmosphere. Percent cytolysis was determined according to the following equation:

$$\%\ \text{cytotoxicity} = \frac{\text{Test cpm} - \text{spontaneous release cpm}}{\text{Total cpm} - \text{spontaneous release cpm}} \times 100.$$

for any of the cell lines tested. Maximum cytolysis was 16.7% for LR3 at E:T ratios of 10:1 and 14% at 20:1. There was no distinct E:T dose-response for any of the cell lines, thus the cytolysis observed was probably nonspecific. In other studies (Aeed *et al.*, 1988) the maximum PMN-mediated cytolysis of 13762NF mammary adenocarcinoma cells was 7.5% in poorly metastatic clone at E:T of 100:1. Stimulation of PMN with PMA did not increase their ability to cytolyse 13762NF mammary adenocarcinoma cell clones or locally recurrent tumour cell lines (data not shown).

### *Macrophage cytolysis*

There was no appreciable cytolysis using unstimulated macrophages at any E:T or co-incubation time (data not shown) which also confirms previously reported observations (North & Nicolson, 1985). Using LPS-activated macrophages, there was generally no macrophage mediated cytolysis of any cells at incubation times of 24 and 48 h (Table II). At 72 h, however, MTF7, scl, and LR1 were maximally lysed at 25.1%, 38.7% and 58.8% at E:T ratio of 20:1 (Table II). MTF7 cytolysis sensitivity was similar to that previously described at 72 h and at comparable E:T (North & Nicolson, 1985). LR1 was lysed at a significantly ($P<0.05$) higher level than scl, and both were lysed at significantly higher levels than the 'parental' MTF7 line. Maximal lysis for LR1a, sc3 and LR3 ranged from 0 to 4.4%. Thus, there are locally recurrent lines that are more sensitive (scl and LR1) and more resistant (sc3, LR3 and LR1a) than MTF7 to activated macrophage cytolysis.

**Table II** Syngeneic macrophage cytolysis of locally recurrent 13762NF mammary adenocarcinoma cell lines[a].

| | | *Percent cytolysis* | | |
|---|---|---|---|---|
| | | *Effector: Target cell ratio* | | |
| *Coincubation time (h)* | *Cell line* | *5:1* | *10:1* | *20:1* |
| 24 | MTF7 | 0 | 0 | 0 |
| | scl | 0.1 | 0 | 0 |
| | LR1 | 3.0 | 0 | 0 |
| | LR1a | 4.9 | 0 | 0.5 |
| | sc3 | 0 | 1.2 | 2.5 |
| | LR3 | 0 | 1.3 | 0 |
| 48 | MTF7 | 0 | 0.7 | 0.4 |
| | scl | 0 | 0 | 0 |
| | LR1 | 4.8 | 0 | 0 |
| | LR1a | 4.9 | 0 | 0 |
| | sc3 | 0 | 0.9 | 0 |
| | LR3 | 0 | 0 | 0 |
| 72 | MTF7 | 14.6 | 12.7 | 17.4 |
| | scl | 21.9 | 23.8[b] | 38.7[b] |
| | LR1 | 40.7[b,c] | 40.3[b,c] | 58.8[b,c] |
| | LR1a | 0[b,c,d] | 0[b,c,d] | 0[b,c,d] |
| | sc3 | 0[b] | 1.8[b] | 3.0[b] |
| | LR3 | 2.8[b] | 4.4[b] | 6.3[b] |

[a]$1 \times 10^4$ [$^{125}$I]UdR labeled tumour cells were co-incubated with LPS-activated for 24h at 37°C in a humidified atmosphere. Label released was used to determine specific cytolysis according to the following equation:

$$\%\ \text{cytotoxicity} = \frac{\text{Test cpm} - \text{spontaneous release cpm}}{\text{Total cpm} - \text{spontaneous release cpm}} \times 100;$$

[b]Significantly different ($P<0.05$) from MTF7 at the same E:T ratio using Student's *t*-test; [c]Significantly different ($P<0.05$) from scl at the same E:T ratio; [d]Significantly different ($P<0.05$) from LR1 at the same E:T ratio.

### *NK cytolysis*

Most of the locally recurrent mammary tumours are not lysed by naive, syngeneic NK cells at E:T up to 100:1 (Figure 2). Maximal cytolysis was 4% for LR3 and the range for all locally recurrent cell lines was 0.5–4.0%. If a nonspecific immunomodulator, bropirimine, is used to activate NK cells prior to isolation (Lotzova *et al.*, 1983), there is a slight increase in the ability of NK cells to cause 13762NF mammary adenocarcinoma cell killing, mostly at the highest E:T levels. ABPP-elicited NK cells caused up to 28% killing for sc3, but only at E:T of 100:1. Killing was well under 20% for all of the other LR sublines and parents at all of the E:T tested. In contrast, bropirimine-activated NK cells are able to kill 50% to almost 100% of YAC-1A cells at low (20:1) effector to target ratios (Figure 1 & Table III). In separate experiments, E:T ratios up to 50:1 with the potent NK cell activator IL-2 alone or in combination with bropirimine resulted in a maximum tumour cell kill of 17% (Table III). Use of NK cells activated with combinations of bropirimine and IL-2 were no more effective at killing 13762NF mammary adenocarcinoma cells or YAC-1a cells than either drug alone for the most part (Table III).

### Discussion

It is now well accepted that most solid tumours are comprised of a heterogeneous mixture of cells which differ for multiple phenotypes (Heppner, 1984). Likewise, it is becoming increasingly clear that tumour composition changes during the course of tumour growth and progression (Neri & Nicolson, 1981; and reviewed in Welch & Tomasovic, 1985; Nicolson, 1987). The implications of dynamic heterogeneity have been discussed and widely studied; however, the mechanisms involved in controlling tumour diversification and tumour composition are still unknown. Likewise, the total

**Figure 2** Sensitivity of 13762NF rat mammary adenocarcinoma locally recurrent cell lines and clones to ABPP-stimulated NK cytolysis *in vitro*. Rats were injected i.p. three days prior to NK cell harvest with SV100 containing varying concentrations of ABPP. Cytolysis was determined by measuring cpm released from [$^{125}$I]-UdR-labeled tumour cells in 96-well microtitre plates. Bars represent mean of quadruplicate samples (s.e.m. is <5% for all points). Open bars (□) represent E:T of 10:1; slashed bars (▧), E:T=20:1; solid bars (■), E:T=50:1; and cross-hatched bars (▩), E:T=100:1. Please note different scale for YAC-1A cells compared to rat adenocarcinoma cell lines.

impact of changing tumour composition is still being determined. It is already known that the generation and maintenance of heterogeneity are both tumour cell directed and tumour cell response to external environment (Welch, 1987, 1988; Welch & Tomasovic, 1985). Neoplastic cells are extremely unstable and generate variants at high rates in what appears to be a preprogrammed manner (reviewed in Welch & Tomasovic, 1985). The composition of a tumour is regulated by interactions with host cells and selective pressures which eliminate some variants spontaneously generated by tumour subpopulations.

The most obvious interaction (i.e. selective pressure) between neoplastic cells and normal cells occurs with the immune system (Fidler & Kripke, 1980). Immune cells respond to subsets of tumour cells and eliminate immunogenic portions of the tumour mass. Besides host selective pressures, artificial selective pressures (i.e. therapy) can also reduce tumour mass. One could predict differences between tumours analyzed prior to and after therapy and that the latter would be more resistant to the same follow-up treatment (Goldie & Coldman, 1984). Immune cells also are involved in changing properties of tumour cells by nontoxic mechanisms. For example, heterotypic embolus formation (Liotta *et al.*, 1976, Fidler *et al.*, 1979), use of enzymes secreted by immune cells (Aeed *et al.*, 1988; Dabbous *et al.*, 1986) and undetermined mechanisms are involved in changing metastatic potential (Fidler *et al.*, 1979). Therefore, these studies were designed to address the connection, if any, between immune cell responsiveness before and after surgery (well after effects of drugs, anaesthetic etc. were past). Since locally recurrent tumours often differ in malignant potentials and sensitivities to follow-up therapy, we hypothesized that some of the changes may be related, in part, to changes in sensitivity to immune cell killing.

Cancer therapy currently involves debulking (often with surgery, but also with radiation, chemotherapy and/or immunotherapy) and 'mopping up' with radiation therapy, chemotherapy or immunotherapy. Drift of tumour populations indicates that follow-up therapy may need to account for shifting populations or different proportions of subpopulations than were present in the primary tumour, therefore changes in sensitivities to individual treatment arms would be observed. Our previous results confirmed this hypothesis since locally recurrent tumour-derived sublines were apparently randomly assorted from the primary tumour for metastatic potentials (Estrada *et al.*, 1986) and commonly used chemotherapy and radiation therapy approaches (Welch *et al.*, 1986; 1988). There was some predictability in the phenotypic drift since many recurrences were indeed more resistant to 5-fluoro-2′-deoxyuridine (at the $LD_{90}$ dose) compared to the primary tumours (Welch *et al.*, 1988). Also, there were some common shifts in 2-dimensional and cell surface protein patterns for all of the recurrences compared to the primary (MTF7) cell line, in particular was the progressive loss of a $M_r \sim 93{,}000$ kDa sialoglycoprotein in all of the recurrences (Welch *et al.*, 1988). Besides selection with artificial 'selective pressures' we also wanted to determine whether recurrent tumours were more or less susceptible to nonspecifically activated host immune cells. As a corollary, we wanted to address a basic question: are nonspecifically activated (i.e. by therapeutic intervention) immune cells effective against all target cells?

13762NF mammary adenocarcinoma locally recurrent tumours regrew after significant surgical elimination of a large part of the tumour mass. Syngeneic F344/NHsd rats mounted an immune response to the primary MTF7 tumour but it was inadequate to prevent tumour growth. Several local recurrences elicited marked immune infiltration as well, but there was significant heterogeneity in the host response (Estrada *et al.*, 1986). Leukocyte infiltration generally appeared to increase in recurrences compared to the primary tumour in most cases; however, some tumour lines elicited less infiltration. And there was also evidence of zonal heterogeneity in host response which was apparent in different histologic sections.

Several immune cells have been shown to play a role in the metastatic properties of tumour cells (Fidler *et al.*, 1979; Hanna, 1982, 1985; Gorelik *et al.*, 1982; Glaves, 1983;

**Table III** Cytolysis of IL-2- and ABPP-stimulated NK cells on locally recurrent rat mammary adenocarcinoma cell lines[a].

| *Treatment* | | | *Percent cytolysis* | | | | | | |
|---|---|---|---|---|---|---|---|---|---|
| *ABPP* | *IL-2(IU ml⁻¹)* | *E:T*[b] | *MTF7* | *scl* | *LR1* | *LR1a* | *sc3* | *LR3* | *YAC-1a* |
| – | — | 0:1 | 1 | 0 | 0 | 1 | 1 | 2 | 3 |
| | | 20:1 | 3 | 0 | 1 | 1 | 1 | 11 | 23 |
| | | 50:1 | 6 | 1 | 0 | 0 | 1 | 10 | 26 |
| – | 0.30 | 0:1 | 1 | 0 | 0 | 0 | 1 | 6 | 4 |
| | | 20:1 | 2 | 1 | 1 | 1 | 3 | 10 | 27 |
| | | 50:1 | 6 | 1 | 1 | 0 | 3 | 12 | 28 |
| – | 3.00 | 0:1 | 2 | 0 | 0 | 0 | 0 | 5 | 3 |
| | | 20:1 | 5 | 1 | 1 | 1 | 2 | 10 | 28 |
| | | 50:1 | ND[c] | ND | ND | ND | ND | ND | ND |
| – | 30.0 | 0:1 | 0 | 0 | 0 | 1 | 1 | 7 | 1 |
| | | 20:1 | 0 | 1 | 1 | 1 | 2 | 7 | 12 |
| | | 50:1 | 2 | 2 | 1 | 1 | 2 | 11 | 53 |
| + | — | 0:1 | 1 | 0 | 0 | 0 | 0 | 7 | 4 |
| | | 20:1 | 1 | 1 | 1 | 1 | 2 | 9 | 25 |
| | | 50:1 | 2 | 9 | 4 | 2 | 11 | 14 | 49 |
| + | 0.30 | 0:1 | 3 | 0 | 0 | 0 | 0 | 5 | 3 |
| | | 20:1 | 3 | 1 | 1 | 1 | 2 | 9 | 27 |
| | | 50:1 | 5 | 9 | 12 | 3 | 11 | 15 | 38 |
| + | 3.00 | 0:1 | 4 | 0 | 0 | 0 | 0 | 7 | 7 |
| | | 20:1 | 1 | 8 | 3 | 1 | 1 | 10 | 25 |
| | | 50:1 | 2 | 7 | 2 | 5 | 11 | 15 | 38 |
| + | 30.0 | 0:1 | 2 | 0 | 0 | 0 | 0 | 6 | 3 |
| | | 20:1 | 0 | 1 | 1 | 2 | 0 | 8 | 30 |
| | | 50:1 | 1 | 8 | 7 | 4 | 9 | 17 | 50 |

[a]1 × 10⁴ [¹²⁵I]UdR-labeled tumour cells were co-incubated with NK cells activated with 200 mg kg⁻¹ ABPP i.p. *in vivo* 3 days prior to harvest, *in vitro* with IL-2, or both for 4 h at 37°C. Percent cytolysis was determined by the following formula:

$$\%\ \text{cytotoxicity} = \frac{\text{Test cpm} - \text{spontaneous release cpm}}{\text{Total cpm} - \text{spontaneous release cpm}} \times 100;$$

[b]Effector to target cell ratio; [c]ND, not determined.

Dabbous *et al.*, 1986; Aeed *et al.*, 1988). Effects can be either stimulatory or inhibitory depending upon immune cell type and tumour cell type (Fidler *et al.*, 1979). Hanna and colleagues have shown that NK cells are highly efficient at clearing tumour cells from the circulation and susceptibility of tumour cells to NK-mediated killing is often, but not always, inversely correlated with metastatic potential (Hanna, 1982; 1985). In the 13762NF mammary adenocarcinoma system none of the cells are susceptible to NK killing at high E:T ratios even when NK activity is enhanced by the immunomodulators bropirimine and/or interleukin-2. Despite possessing highly heterogeneous metastatic potentials, NK resistance of LR cell lines is identical, indicating that this is not a property correlative of metastatic potential in the 13762NF mammary adenocarcinoma locally recurrent tumour model system. Similarly, NK susceptibility does not change as recurrent sublines are selected from primary tumour growths.

It is interesting to note that administration of bropirimine to rats 3 days prior to NK harvest or addition of interleukin-2 to the culture medium resulted in marked activation of NK cells for cytolysis of YAC-1A cells. There was also a weak, dose-dependent increase in 13762NF mammary adenocarcinoma cell killing, but this was unremarkable (Figure 2 & Table III). Maximal cytolysis for mammary tumour cells was 28% (in only one experiment) compared to 50–>90% for the YAC-1A cell line. This demonstrates that, despite complete activation, NK cells do not become capable of eliminating ALL tumour cells, especially those resistant initially.

Gorelik *et al.* (1982) showed that co-injection of macrophages with B16 melanoma or Lewis lung carcinoma (3LL) tumour cells resulted in increased pulmonary colonization. Similarly, Starkey *et al.* (1984) showed that heterotypic aggregates containing PMN or macrophages and rat hepatocarcinoma cells were more capable of pulmonary colonization than tumour cells alone. These data show that tumour cells cooperate with some immune cells to enhance properties important for metastatic colonization. On the other hand, Fidler, Poste & colleagues have examined the role of activated macrophages in the prevention and elimination of B16 melanoma metastases (Fidler *et al.*, 1985; Poste, 1984). They have found that liposome-activated macrophages (muramyl dipeptide-containing liposomes) are capable of preventing and eradicating established micrometastases. North & Nicolson (1985) found that there was no correlation between metastatic potential of 13762NF mammary adenocarcinoma cell clones and sensitivity to LPS-activated-macrophage-mediated cell lysis, intratumoural macrophages or thioglycolate-elicited macrophages. Our conclusions are similar with the recently derived locally recurrent tumour model. MTF7 and LR3 form approximately the same number lung colonies (>200 metastases per rat (Estrada *et al.*, 1986) yet 20–25% of MTF7 *versus* 0–4% of LR3 cells are killed in a macrophage cytolysis assay. scl and sc3 form approximately one-half as many lung colonies as MTF7 but macrophage killing occurs for 22–38% and 0–3% of the cells, respectively. Likewise, there is no pattern towards increased or decreased sensitivity to macrophage killing in the local recurrence lineages.

Polymorphonuclear cells make up a large percentage of the circulating leukocytes in human blood; however, PMN account for only 10–20% of circulating white blood cells in F344/NHsd rats. We have shown that PMN levels in the blood rise sharply in 13762NF mammary adenocarcinoma tumour-bearing rats and the increase is proportional to the metastatic potential of the tumour. The circulating neutrophils secrete high levels of collagenase IV and a heparan sulphate endoglycosidase suggesting that they may be assisting tumour cell extravasation (Aeed *et al.*, 1988). Glaves (1983) found that activation of PMN in the vasculature with trypan dye injection resulted in increased oxygen radical production and increased radiolabeled tumour cell clearance following intravenous inoculation. She proposed that PMN may be important in limiting metastatic potential by eliminating circulating B16 melanoma cells. While PMN in our experiments are completely active in that they respond to PMA and FMLP to degranulate or produce oxygen metabolites, they are essentially unable to kill 13762NF mammary adenocarcinoma locally recurrent tumour cells in the presence or absence of these agents. Others have shown that PMN are capable of killing tumour cells (Pickaver *et al.*, 1972; Kondo *et al.*, 1986; Lichenstein, 1987; Lichenstein & Kahle, 1985; Morikawa *et al.*, 1985). As with NK cells and macrophages, there was no trend towards increased resistance with the local recurrent lineages tested.

All of the results presented here are the result of interactions between a single immune cell type with tumour cells. Lack of cytotoxicity with NK or PMN may have limited meaning since there may be synergy *in vivo*. The results do, within these limits, allow direct comparison of sensitivities under the conditions described and the sensitivities are significantly different. Extrapolation to the complex situation in an immunocompetent host is difficult.

In summary, locally recurrent tumours differ significantly from the primary tumour for a variety of properties, including sensitivity to natural immune mechanisms. Some recurrences were significantly more sensitive to macrophage mediated cell killing while others were significantly more resistant. None of the 13762NF mammary adenocarcinoma sublines were sensitive to activated NK or PMN cell killing. And there was no apparent correlation in the sensitivity of a single cell line to one immune cell type to another immune cell type. These results imply that changes in cellular composition of locally recurrent tumours may overwhelm the body's ability to adapt to and to mount an effective immune response against all survivors. They do not imply that all of the recurrences are resistant to immune cell killing. Some, in fact, may be more sensitive. Our results do demonstrate that follow-up therapy for recurrent tumours, even treatment with nonspecific immunomodulators, must account for changes in tumour composition.

We gratefully acknowledge the assistance of Dr R. Smith, L.M. Sam and J.M. Justen for assistance with PMN function assays; Dr L.H. Li and T.L. Wallace for assistance with NK cytolysis assays and helpful comments; R. Howrey, P. Wack and J. Mantia for technical assistance; Dr D. Branstetter for histological analysis of LR tumours and C.A. Kiewiet for secretarial assistance.

### References

AEED, P.A., NAKAJIMA, M. & WELCH, D.R. (1988). The role of polymorphonuclear leukocytes (PMN) on the growth and metastatic potential of 13762NF mammary adenocarcinoma cells. *Int. J. Cancer* (in press).

CHEN, T.R (1977). *In situ* detection of mycoplasma contamination in cell cultures by fluorescent Hoechst 33258 stain. *Exp. Cell Res.*, **104**, 255.

CHUN, M., KRIM, M., GRANELLI-PIPERNA, A., HIRST, J.A. & HOFFMAN, M.K. (1985). Enhancement of cytotoxic activity of natural killer cells by interleukin and antagonism between interleukin 2 and adenosine cyclic monophosphate. *Scand. J. Immunol.*, **22**, 375.

DABBOUS, M.KH., WOOLEY, D.E., HANEY, L., CARTER, L.M. & NICOLSON, G.L. (1986). Host-mediated effectors of tumour invasion: Role of mast cells in matrix degradation. *Clin. Exp. Metastasis*, **4**, 141.

DONNEGAN, W.L., PEREZ-MESA, C.M. & WATSON, F.R. (1966). A biostatistical study of locally recurrent breast carcinoma. *Surg. Gyn. Obst.*, **122**, 529.

ESTRADA, J., FREEMAN, D.H., AEED, P.A. & WELCH, D.R. (1986). Characterization of locally recurring mammary tumours: Differences in growth kinetics and metastatic potential. *Proc. Am. Assoc. Cancer Res.*, **27**, 61 (*abstract*).

FIDLER, I.J. (1970). Quantitative analysis of distribution and fate of tumour emboli labeled with [$^{125}$I]-5-iodo-2′-deoxyuridine. *J. Natl Cancer Inst.*, **45**, 773.

FIDLER, I.J., FOGLER, W.E., KLEINERMAN, E.S. & SAIKI, I. (1985). Abrogation of species specificity for activation of tumoricidal properties in macrophages by recombinant mouse or human interferon-gamma encapsulated in liposomes. *J. Immunol.*, **135**, 4289.

FIDLER, I.J., GERSTEN, D.M. & KRIPKE, M.L. (1979). Influence of immune status on the metastasis of three murine fibrosarcomas of different immunogenicities. *Cancer Res.*, **39**, 3816.

FIDLER, I.J. & KRIPKE, M.L. (1980). Tumour cell antigenicity, host immunity and cancer metastasis. *Cancer Immunol. Immunother.*, **7**, 201.

FISHER, B., GLASS, A., REDMAN, C. & 14 others (1977). L-phenylalanine mustard (L-PAM) in the management of primary breast cancer: An update of earlier findings and a comparison with those utilizing L-PAM plus 5-fluorouracil (5-FU). *Cancer*, **39**, 2283.

GLAVES, D. (1983). Role of polymorphonuclear leukocytes in the pulmonary clearance of arrested cancer cells. *Invasion Metastasis*, **3**, 160.

GOLDIE, J.H. & COLDMAN, A.J. (1984). The genetic orifin of drug resistance in neoplasms. Implications for systemic therapy. *Cancer Res.*, **44**, 3643.

GORELIK, E., SEGAL, S., SHAPIRO, J., KATZAV, S., RON, Y. & FELDMAN, M. (1982). Interactions between the local tumor and its metastases. *Cancer Metastasis Rev.*, **1**, 83.

GORELIK, E., WILTROUT, R.H., BRUNDA, M.J., HOLDEN, H.T. & HERBERMAN, R.B. (1982). Augmentation of metastasis formation by thioglycolate-elicited macrophages. *Int. J. Cancer*, **29**, 575.

HANNA, N. (1982). Role of natural killer cells in control of cancer metastasis. *Cancer Metastasis Rev.*, **1**, 45.

HANNA, N. (1985). The role of natural killer cells in the control of tumor growth and metastasis. *Biochim. Biophys. Acta.*, **780**, 213.

HEPPNER, G.H. (1984). Tumor heterogeneity. *Cancer Res.*, **44**, 2259.

HERSH, E.M., GUTTERMAN, J.U., MAVLIGET, G.M. & MURPHY, S.G. (1980). Current status of human cancer immunotherapy. In *Advances in Immunopharmacology*, Hadden, J. *et al.* (eds) p. 227. Pergamon Press: Elmsford, New York.

KARABALI-DALAMAGA, B., SOUHAMI, R.L., O'HIGGINS, N.J., SOUMILAS, A. & CLARK, C.G. (1978). Natural history and prognosis of recurrent breast cancer. *Br. J. Med.*, **2**, 730.

KONDO, M., HARUKI, K., YOSHIKAWA, T. & SUGINO, S. (1986). Treatment of cancer ascites by intraperitoneal administration of a streptococcal preparation of OK-432 with fresh human complement – role of complement-derived chemotactic factor to neutrophils. *Int. J. Immunopharmac.*, **8**, 715.

LI, L.H., DEKONING, T.F. & WALLACE, T.L. (1987). Relationship between modulation of natural killer cell activity and antitumor activity of bropirimine when used in combination with various types of chemotherapeutic drugs. *Cancer Res.*, **47**, 5894.

LICHTENSTEIN, A. (1987). Stimulation of the respiratory burst of murine peritoneal inflammatory neutrophils by conjugation with tumour cells. *Cancer Res.*, **47**, 2211.

LICHTENSTEIN, A. & KAHLE, J. (1985). Antitumor effect of inflammatory neutrophils: Characteristics of *in vivo* generation and *in vitro* tumor cell lysis. *Int. J. Cancer*, **35**, 121.

LIOTTA, L.A., KLEINERMAN, E.J. & SARDEL, G.M. (1976). The significance of hematogenous tumor cell clumps in the metastatic process. *Cancer Res.*, **36**, 889.

LOTZOVA, E., SAVARY, C.A. & STRINGFELLOW, D.A. (1983). Modulation of murine NK cell cytoxicity *in vitro* and antitumor activity *in vivo* by low molecular weight interferon inducers. In *Cancer: Etiology and Prevention*, Crispan, R.G. (ed) p. 199. Elsevier/North-Holland Biomedical Press: Amsterdam.

MORIKAWA, K., KAMEGAYA, S., YAMAZAKI, M. & MIZUNO, D. (1985). Hydrogen peroxide as a tumoricidal mediator of murine polymorphonuclear leukocytes induced by a linear -1,3-D-glucan and some other immunomodulators. *Cancer Res.*, **45**, 3482.

NERI, A. & NICOLSON, G.L. (1981). Phenotypic drift of metastatic and cell-surface properties of mammary adenocarcinoma cell clones during growth *in vitro*. *Int. J. Cancer*, **28**, 731.

NERI, A., WELCH, D.R., KAWAGUCHI, T. & NICOLSON, G.L. (1982). The development and biologic properties of malignant cell sublines and clones of a spontaneously metastasizing rat mammary adenocarcinoma. *J. Natl Cancer Inst.*, **68**, 507.

NICOLSON, G.L. (1987). Tumor cell instability, diversification and progression to the metastatic phenotype: From oncogene to oncofetal expression. *Cancer Res.*, **47**, 1473.

NORTH, S.M. & NICOLSON, G.L. (1985). Heterogeneity in the sensitivities of the 13762NF rat mammary adenocarcinoma cell clones to cytolysis mediated by extra- and intratumoral macrophages. *Cancer Res.*, **45**, 1453.

PATANAPHAN, V., SALAZAR, O.M. & POUSSIN-ROSILLO, H. (1984). Prognosticators in recurrent breast cancer. A 15-year experience with irradiation. *Cancer*, **54**, 228.

PEARLMAN, N.W. & JOCHIMSEN, P.R. (1979). Recurrent breast cancer: Factors influencing survival, including treatment. *J. Surg. Oncol.*, **11**, 21.

PICKHAVER, A.H., RATCLIFFE, N.A., WILLIAMS, A.E. & SMITH, H. (1972). Cytotoxic effects of peritoneal neutrophils on a syngeneic rat tumour. *Nature New Biol.*, **235**, 186.

POSTE, G. (1984). Drug targeting in cancer therapy. In *Receptor mediated Targeting of Drugs*, Gregoriadis, G. *et al.* (eds) p. 427. Plenum Press: Philadelphia.

RECHT, A., SILVER, B., SCHNITT, S., CONNOLLY, J., HELLMAN, S. & HARRIS, J.R. (1985). Breast relapse following primary radiation therapy for early breast cancer. I. Classification, frequency and salvage. *Int. J. Radiat. Oncol. Biol. Phys.*, **11**, 1271.

STARKEY, J.R., LIGGITT, H.D., JONES, W. & HOSICK, H.L. (1984). Influence of migratory blood cells on the attachment of tumor cells to vascular endothelium. *Int. J. Cancer*, **34**, 535.

TOONKEL, L.M., FIX, I., JACOBSON, L.H. & WALLACH, C.B. (1983). The significance of local recurrence of carcinoma of the breast. *Int. J. Radiat. Oncol. Biol. Phys.*, **9**, 33.

WELCH, D.R. (1987). Biologic considerations for drug targeting in cancer patients. *Cancer Treatment Rev.*, **14**, 351.

WELCH, D.R. (1988). Factors involved in the development and maintenance of tumor heterogeneity. In *Carcinogenesis and Dietary Fat*, Abraham, S. (ed) (in press). Martinus Nijhoff: New York.

WELCH, D.R., AEED, P.A. & ESTRADA, J. (1986). Characterization of locally recurring mammary tumors: Sensitivities to FUdR and adriamycin. *Proc. Am. Assoc. Cancer Res.*, **27**, 67 (*abstract*).

WELCH, D.R., AEED, P.A. & ESTRADA, J. (1988). Development and characterization of a rat model for locally recurring mammary tumours: Sensitivities to 5-fluoro-2′-deoxyuridine, adriamycin and X-irradiation. *Cancer Res.*, **48**, 4549.

WELCH, D.R., McCLURE, S.A., BAHNER, M.J., AEED, P.A. & ADAMS, L.D. (1988, submitted for publication). Preliminary protein and cell surface analysis of locally recurring mammary tumor cell lines.

WELCH, D.R. & TOMASOVIC, S.P. (1985). The implications of tumor progression on clinical oncology. *Clin. Exp. Metastasis*, **3**, 151.

WIERENGA, W., SKULNICK, H.I., STRINGFELLOW, D.A., WEED, S.D., RENIS, H. & EDISON, E.E. (1980). 5-substituted 2-amino-6-phenyl-4(3H)-pyrimidinones. Antiviral and interferon-inducing agents. *J. Med. Chem.*, **23**, 237.